# Evaluation of a FRET-Peptide Substrate to Predict Virulence in *Pseudomonas aeruginosa*

**DOI:** 10.1371/journal.pone.0081428

**Published:** 2013-11-26

**Authors:** Wendy E. Kaman, Nora El Arkoubi-El Arkoubi, Sanne Roffel, Hubert P. Endtz, Alex van Belkum, Floris J. Bikker, John P. Hays

**Affiliations:** 1 Department of Medical Microbiology and Infectious Diseases, Erasmus Medical Center, Rotterdam, The Netherlands; 2 Department of Oral Biochemistry, Academic Centre for Dentistry Amsterdam, University of Amsterdam and VU University Amsterdam, Amsterdam, The Netherlands; 3 Research & Development Microbiology, BioMérieux, La Balme les Grottes, France; University of Malaya, Malaysia

## Abstract

*Pseudomonas aeruginosa* produces a number of proteases that are associated with virulence and disease progression. A substrate able to detect *P. aeruginosa*-specific proteolytic activity could help to rapidly alert clinicians to the virulence potential of individual *P. aeruginosa* strains. For this purpose we designed a set of *P. aeruginosa*-specific fluorogenic substrates, comprising fluorescence resonance energy transfer (FRET)-labeled peptides, and evaluated their applicability to *P. aeruginosa* virulence in a range of clinical isolates. A FRET-peptide comprising three glycines (3xGly) was found to be specific for the detection of *P. aeruginosa* proteases. Further screening of 97 *P. aeruginosa* clinical isolates showed a wide variation in 3xGly cleavage activity. The absence of 3xGly degradation by a *lasI* knock out strain indicated that 3xGly cleavage by *P. aeruginosa* could be quorum sensing (QS)-related, a hypothesis strengthened by the observation of a strong correlation between 3xGly cleavage, LasA staphylolytic activity and pyocyanin production. Additionally, isolates able to cleave 3xGly were more susceptible to the QS inhibiting antibiotic azithromycin (AZM). In conclusion, we designed and evaluated a 3xGly substrate possibly useful as a simple tool to predict virulence and AZM susceptibility.

## Introduction

*Pseudomonas aeruginosa*, is a gram-negative rod-shaped bacterium, which is an important cause of infection in individuals suffering from a wide range of underlying disease conditions, including individuals with a compromised host defence, burn patients and in patients suffering from the genetically inherited respiratory tract disease cystic fibrosis (CF). *P. aeruginosa* is also a frequent cause of hospital acquired pneumonia, wound infections and bacteraemia [1]. Early detection of a P. *aeruginosa* infection facilitates effective antimicrobial treatment, reduces inappropriate antibiotic prescription and could possibly contribute to preventing irreversible lung disease in CF patients. Additionally, this bacterial pathogen produces a number of protease enzymes that are associated with virulence and disease progression [2]. In this respect, a substrate able to detect *P. aeruginosa*-specific proteolytic activity could help to rapidly alert clinicians to the virulence potential of individual *P. aeruginosa* strains isolated from different patients. Specifically, detection of these protease virulence factors could potentially be used to monitor the severity of infection and to predict disease outcome, thereby providing useful information to support tailor-made patient monitoring and treatment, a first step towards introducing “personalized medicine”. 

In theory, bacterial proteases may be suited as biomarkers for the rapid and sensitive identification of microorganisms in clinical samples. Further, the ability to utilize protease based detection methods to diagnose bacterial infections has been previously described [3,4]. In this respect, it is known that *P. aeruginosa* is equipped with a large arsenal of virulence factors that aid to successfully infect the host [2]. The majority of these virulence factors include proteases produced under control of the Las and Rhl quorum sensing (QS) systems of *P. aeruginosa* [5], examples of which include LasA and LasB elastases and alkaline protease [6]. These proteases play an important role in *P. aeruginosa* pathogenesis through the degradation of biologically active proteins present in human tissue [7]. In fact, the significant role of the QS-system in *P. aeruginosa*-related disease progression has resulted in much research into QS-inhibiting compounds that could potentially reduce the organism’s virulence potential [8–12]. Further, the therapeutic efficacy of this anti-QS, anti-virulence, strategy in the treatment of *P. aeruginosa* has already been demonstrated for the QS-inhibiting antibiotic azithromycin [12]. Though anti-virulence treatment based on QS-inhibition is only useful when an active, virulence factor secreting, QS-system is present in the P. *aeruginosa* strain to be targeted.

In this study we describe the design of a novel *P. aeruginosa*-specific Fluorescence Resonance Energy Transfer (FRET)-peptide substrate and examine its applicability for the detection of *P. aeruginosa* proteolytic activity in clinical specimens. In addition, we studied the link between substrate cleavage efficiency, virulence factor production and susceptibility towards QS-inhibiting antibiotics, such as azithromycin.

## Materials and Methods

### Bacteria

The P. *aeruginosa* strains used for substrate specificity testing are listed in Table 1. Clinical strains of *P. aeruginosa* were collected from a bacterial biobank present within the Department of Medical Microbiology and Infectious Diseases of the Erasmus Medical Center, Rotterdam, Netherlands, collected between the years 2008-2012. In total 97 clinical isolates were selected; 13 from blood, 56 from sputum (35 CF patients and 21 non-CF patients), and 28 strains were isolated from wounds. The VITEK 2 system (bioMérieux, Marcy L`Etoile, France) was used for identification and antibiotic susceptibility testing of the clinical isolates. For the preparation of culture supernatants, all bacteria were grown in 5 ml Brain Heart Infusion (BHI) medium (bioTrading, Mijdrecht, The Netherlands) at 37 °C. After 16 h of culture, the bacteria were pelleted by centrifugation for 10 min at 3000 x g, and the enzyme containing supernatant was filter sterilized through a 0.22 μm filter (Millipore, Amsterdam, The Netherlands).

**Table 1:** Bacterial strains used in this study.

**Strain**	
*Pseudomonas aeruginosa*	ATCC 15692, PAO1
*Pseudomonas aeruginosa*	PA14
*Pseudomonas aeruginosa*	PA14Δ*lasI* [31]
*Pseudomonas fluorescens*	Clinical isolate
*Pseudomonas putida*	S12
*Pseudomonas stutzeri*	DSMZ 10701, JM300
*Staphylococcus aureus*	ATCC 43300
*Staphylococcus simulans*	ATCC 27851
*Staphylococcus capitis* subsp. *capitis*	ATCC 35661
*Staphylococcus epidermidis*	Clinical isolate
*Streptococcus pneumoniae*	ATCC 49619
*Streptococcus equi* subsp. *zooepidemicus*	ATCC 43079
*Klebsiella pneumoniae*	ATCC 43816
*Haemophilus influenzae*	ATCC 49247

### FRET- assay

The substrates used in this study were purchased at PepScan Presto B.V. (Lelystad, The Netherlands) with a purity > 90%. All substrates were C-terminally flanked with a fluorescent probe; FITC and N-terminally flanked with a lysine coupled quencher; Dabcyl. Identity of the substrates was confirmed by PepScan Presto B.V. using mass spectrometry. Assays were performed in black, clear bottom 96-well plates (Corning, Lowell, USA). Proteolytic activity was determined by incubating 16 μM substrate with 50 μl filtered bacterial culture supernatant or 50 µl lysostaphin (0.04 µg/µl diluted in BHI, Sigma, Zwijndrecht, The Netherlands) at 37 °C. Filtered BHI medium was used as a negative control. Plates were read for 60 min with 2 min intervals using a fluorescence microplate reader (FLUOstar Galaxy, BMG Laboratories, Offenburg, Germany) using an excitation wavelength of 485 nm and an emission wavelength of 530 nm. Relative fluorescence (RF) values were obtained after correction against an un-inoculated BHI culture medium control. Protease activity was defined in RF per minute (RF/min).

### Sensitivity testing of the 3xGly substrate *in vitro*

*P. aeruginosa* PAO1 was cultured overnight in BHI medium at 37 °C. Next day, 20 µl of the overnight culture was added to 20 ml fresh BHI medium. Subsequently, bacteria were grown for another 16 h and a sample was taken every hour. The number of bacteria in the sample was determined by colony counting by plating 10-fold serial dilutions on trypticase soy agar (TSA) plates (bioTrading, Mijdrecht, The Netherlands). Plates were incubated at 37 °C and bacteria were enumerated after overnight incubation. Proteolytic activity on the 3xGly substrate was examined using 50 µL samples in the FRET-assay as described above. Relative fluorescence (RF) values were obtained after correction against negative culture medium samples. The protease activity was defined in RF per minute (RF/min). Proteolytic activity with an RF/min > 5 was defined positive.

### Quantification of pyocyanin production and LasA protease activity

Extracellular pyocyanin production was determined as previously described [13]. LasA protease activity was examined by measuring the ability of stationary phase *P. aeruginosa* culture supernatants (see FRET-assay section above) to lyse heat-killed *Staphylococcus aureus* cells as described by Kessler et al [14].

### RNA isolation and cDNA synthesis

A selection of 12 *P. aeruginosa* isolates were chosen for RNA isolation. These isolates had been cultured from blood, wound and sputum clinical specimens, with two isolates that were 3xGly active (+, RF/min > 5) and two isolates that were 3xGly inactive (-, RF/min < 5), being chosen from each clinical specimen type .*P. aeruginosa* strain PAO1 (ATCC 15692) was used as a 3xGly positive reference control. The 12 *P. aeruginosa* isolates were initially grown overnight in BHI medium at 37°C,. Next day the overnight culture was diluted 1:10 in BHI medium and the bacteria again cultured at 37 °C until an OD_600_ of approximately 0.9 - 1.0 was reached. From these cultures total RNA was extracted using the FastRNA ProBlue kit (Promega, Leiden, The Netherlands) according to manufacturer’s instructions. To remove any contaminating DNA, 1 μg of extracted RNA was mixed with one unit DNase (Fermentas, Thermo Fisher, Landsmeer, The Netherlands) in 1x DNase reaction buffer. The mixture was then incubated at 37 °C for 30 min and the reaction stopped by the addition of 1 μl 50 mM EDTA and subsequent incubation for 10 min at 65 °C. For cDNA synthesis, DNase treated RNA (500 ng) was incubated for 60 min at 42 °C with 5 µM random hexamer primers, 10 units Ribolock, 1 mM dNTPs and 200 units RevertAid in 1x reaction buffer (Fermentas, Thermo Fisher, Landsmeer, The Netherlands). The reaction was stopped by incubation at 70 °C for 5 min.

### Expression of quorum sensing genes

The detection of QS-gene expression was performed using real-time PCR amplification and specifically designed primers for the QS-related pathway genes *lasI* and *rhlA*, the non-QS related control gene *trpD* and the P. *aeruginosa* housekeeping gene *rpsL* [15–17]. Each PCR contained 1x FastStart SYBR Green mastermix (Roche, Woerden, The Netherlands), 4 mM MgCl_2_ and 0.5 pmol of each primer and 2 μl of 10x diluted cDNA. Amplification was performed using the LightCycler (Roche, Woerden, The Netherlands) The cycling parameters used were: 15 min at 95 °C, 40 amplification cycles of 95 °C for 20 s, 60 °C for 20 s and 72 °C for 30 s. Melting curve analysis, performed at the end of amplification, showed a single product peak, indicating that no non-specific products were amplified. Data were analyzed using LightCycler software (version 3.0) and the housekeeping gene *rpsL* was used as a reference (housekeeping) gene for normalizing gene expression. The 2^-ΔΔCt^ method was used to calculate the expression of the genes of interest [18]. QS-gene expression was calculated as a relative percentage of the *rpsL*-normalized gene expression in the control *P. aeruginosa* PAO1 isolate.

### Azithromycin susceptibility testing

The susceptibility of *P. aeruginosa* to the QS inhibiting antibiotic azithromycin (AZM) was evaluated for 35 *P. aeruginosa* strains isolated from the sputa of CF-patients using the disc diffusion method. For this purpose, a suspension of each bacterial isolate, overnight grown on tryptic soy agar (TSA) blood agar, was prepared in physiological saline at a turbidity of 0.5 McFarland units. Each bacterial suspension was streaked onto TSA plates with a sterile cotton swab, to obtain uniform bacterial growth, and a disc containing 15 µg AZM (Oxoid, Badhoevedorp, The Netherlands) was placed on the middle of the inoculated culture plate. Plates were then incubated at 37 °C, incubated overnight and the diameter of the zones of growth inhibition (mm) were measured. 

## Results

### Design of *P. aeruginosa*- specific FRET-substrates

**Figure 1:**
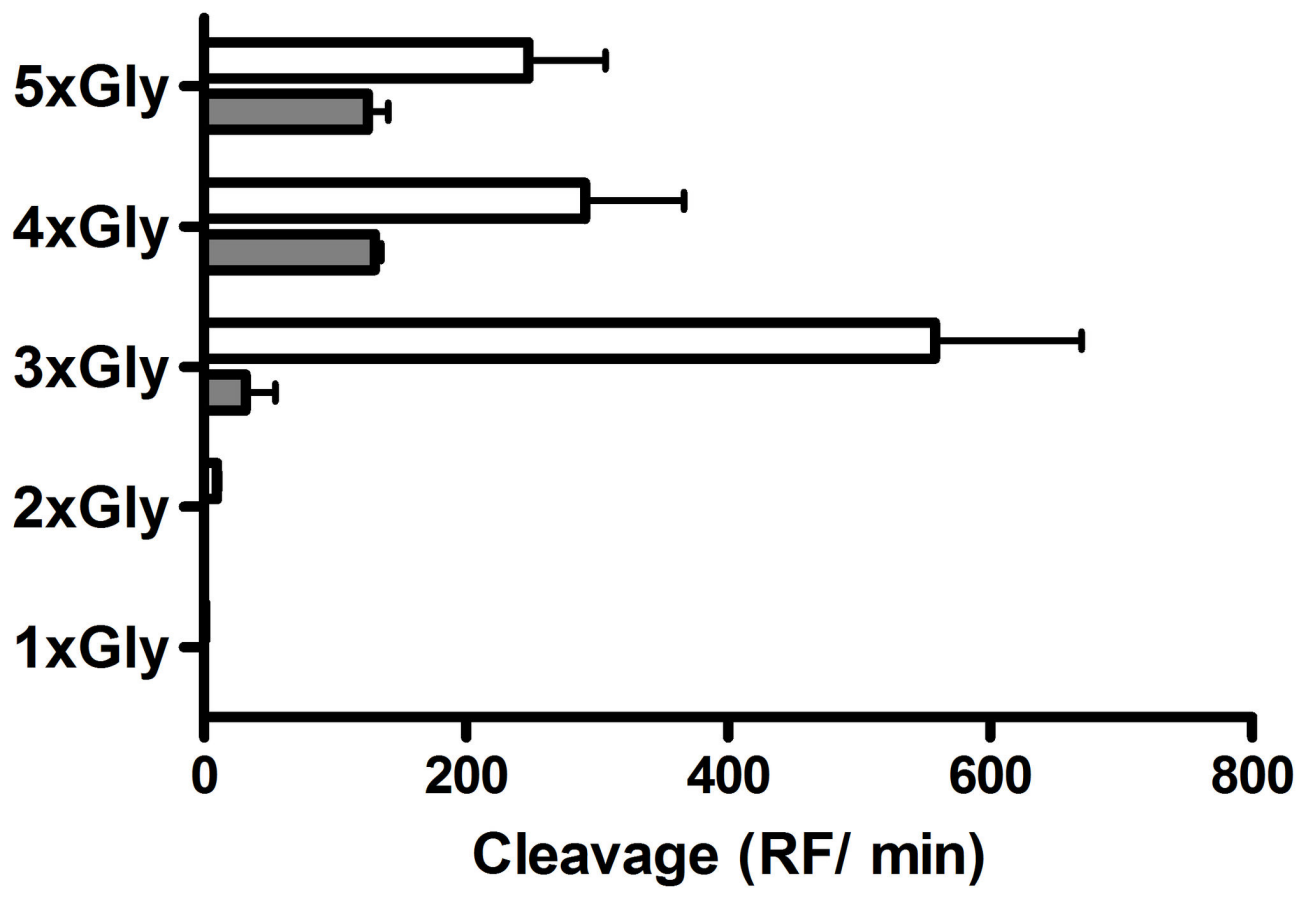
Cleavage activity of lysostaphin and *P. aeruginosa* culture supernatant on a range of glycine substrates. Lysostaphin (2 µg, grey bar) and culture supernatant of *P. aeruginosa* PAO1 (white bar) were incubated with 16 µM FRET-substrate at 37 °C for 1 h. Cleavage of the substrates was defined in Relative Fluorescence per minute (RF/min). Results are expressed as mean ± SEM (n = 3).

In a study by Vessillier et al. it was described that the P. *aeruginosa* specific protease LasA, recognizes and degrades glycine bonds [19]. Based on these cleavage characteristics we designed FRET-peptide substrates, comprising multiple glycine residues, and incubated these substrates with *P. aeruginosa* culture supernatant. These substrates included FITC-Gly-LysDbc (1xGly), FITC-(Gly)_2_-LysDbc (2xGly), FITC-(Gly)_3_-LysDbc (3xGly), FITC-(Gly)_4_-LysDbc (4xGly) and FITC-(Gly)_5_-LysDbc (5xGly). As a specificity control, we compared the activity of *P. aeruginosa* culture supernatant towards these substrates with cleavage activity with lysostaphin, an enzyme produced by *Staphylococcus simulans*, that recognizes and degrades penta-glycine bonds [20]. Experiments showed that the 5xGly substrate was cleaved by both lysostaphin and *P. aeruginosa* culture supernatant, and that a similar result was observed for the substrate containing four glycines (Figure 1). However, when the 3xGly substrate was tested, a significant decrease in proteolytic activity by lysostaphin was observed, whereas the proteolytic activity of *P. aeruginosa* actually significantly increased. Additional experiments showed minimal cleavage in case of the 2xGly substrate and no cleavage using the 1xGly substrate. These results indicated that the 3xGly substrate was the best candidate, amongst the substrates tested, to investigate as a potential substrate for the rapid detection of virulence in *P. aeruginosa*. 

### Characterization of 3xGly substrate cleavage

**Figure 2:**
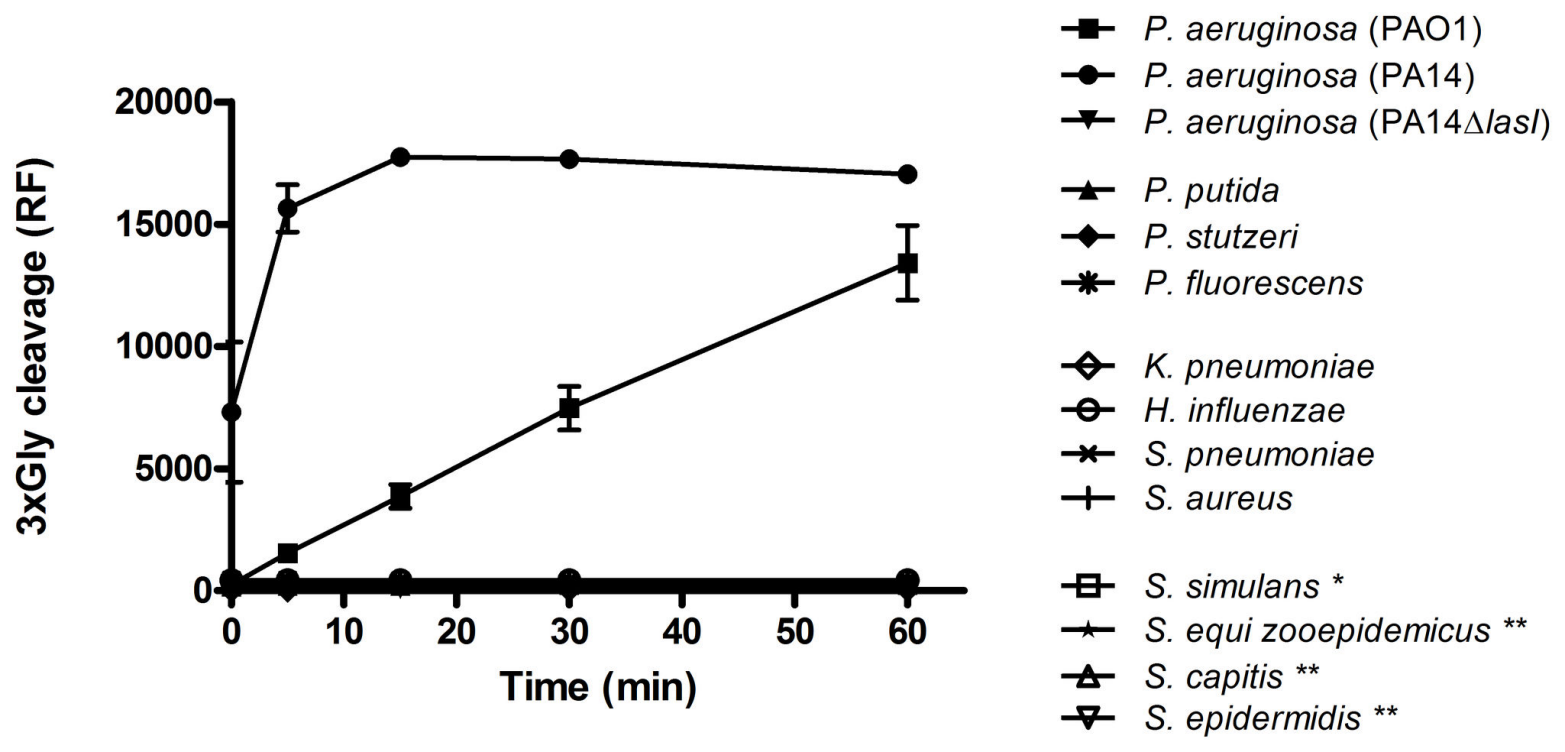
Specificity testing of the 3xGly substrate. Culture supernatants of *Pseudomonas* spp., respiratory microorganisms and bacteria producing lysostaphin (*) or lysostaphin-like (**) proteases were incubated with 16 µM 3xGly. Fluorescence was measured for 1 h at 37 °C. Results are expressed as mean ± SEM (n = 3).

**Figure 3:**
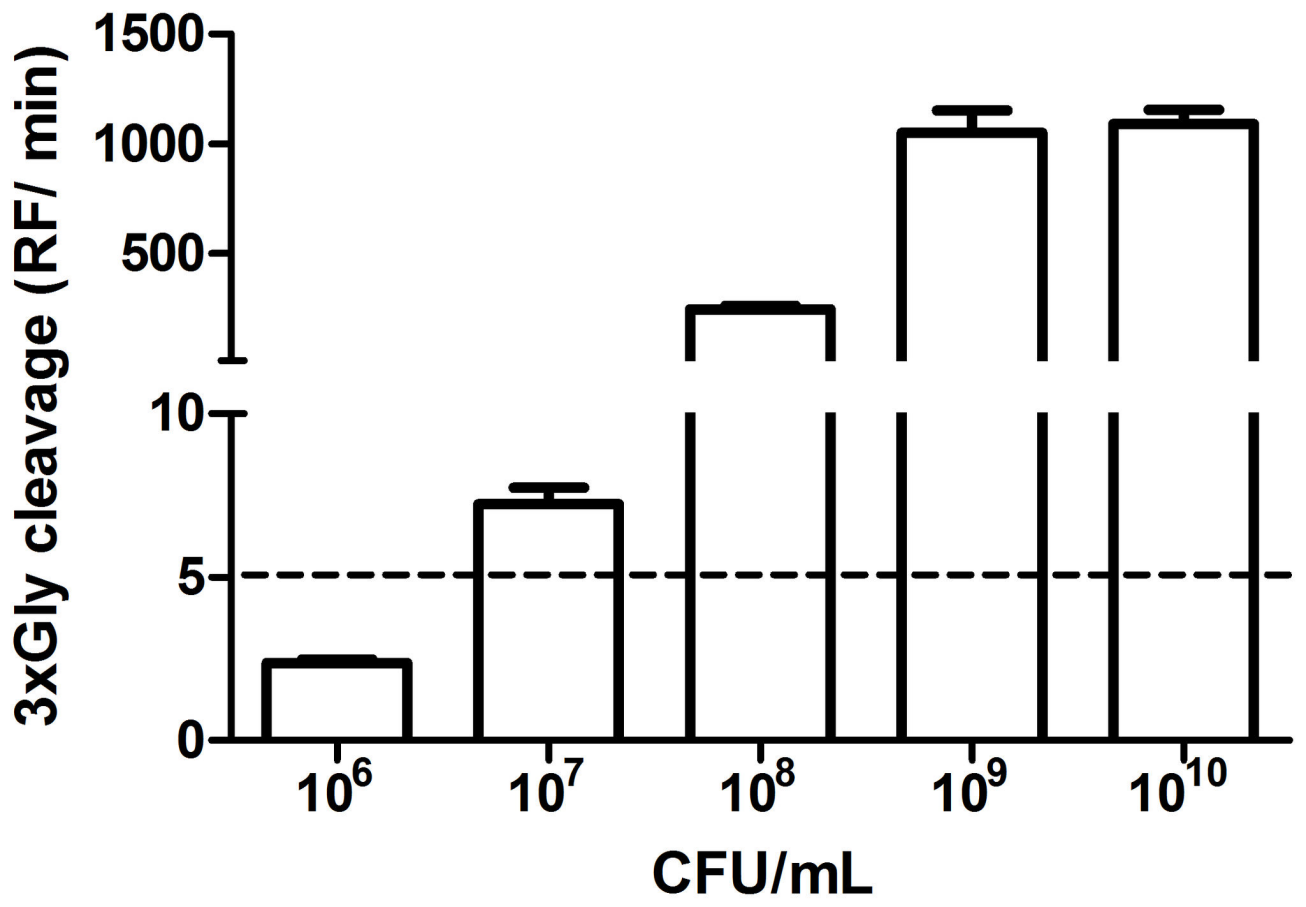
Sensitivity testing of the 3xGly substrate. Serial dilutions of *P. aeruginosa* PAO1 were incubated with 16 µM 3xGly at 37 °C. Cleavage activity was defined in Relative Fluorescence per minute (RF/min). The cut-off of the assay was estimated at an RF/min of 5. Results are expressed as mean ± SEM (n = 3).

To characterize the specificity of the 3xGly substrate, we examined 3xGly cleavage using a range of culture supernatants (i) from a range of *Pseudomonas* non-*aeruginosa* species, and (ii) from a range of additional respiratory bacteria, and microorganisms known to produce lysostaphin (-like) proteases (Table 1). Results showed that the 3xGly substrate was exclusively cleaved by culture supernatants of *P. aeruginosa* (strains PA14 and PAO1), but not by *P. putida*, *P. stutzeri* or *P. fluorescens*. Further, no cleavage activity was observed for the range of additional respiratory bacteria, and microorganisms known to produce lysostaphin, that were tested. Interestingly, no cleavage activity was observed when the 3xGly substrate was incubated with culture supernatant of the P. *aeruginosa* strain PA14∆*lasI* which lacks the *lasI* QS-gene (Figure 2). Besides the above mentioned bacteria the 3xGly substrate was screened with an additional set of in total 17 bacterial supernatants. None of these bacteria was able to cleave the substrate (data not shown). To characterize 3xGly sensitivity, the substrate was incubated with a dilution series of *P. aeruginosa* PAO1. Using a cut-off of RF/min > 5, it was observed that the limit of detection for *P. aeruginosa* PAO1 using the 3xGly substrate was 10^7^ CFU//ml (Figure 3). 

In order to obtain a more comprehensive view of protease activity in an extended range of clinically relevant *P. aeruginosa* isolates, we tested a total of 97 randomly selected *P. aeruginosa* clinical isolates that had been cultured from wounds, blood and sputum. These strains were analyzed for their supernatant cleavage activity on the 3xGly substrate. Results revealed that a large percentage of the strains tested (60/97; 62%) were unable to cleave the 3xGly substrate. This percentage varied between clinical specimens from 50% in wound isolates to 73% in isolates from CF patients (Table 2). 

**Table 2:** 3xGly cleavage activity among 97 randomly selected *P. aeruginosa* clinical isolates ^***a***^.

			**Sputum**	
			(*n*=56)	
	**Wound**	**Blood**	**CF**	**Non-CF**
	(*n*=28)	(*n*=13)	(*n*=35)	(*n*=21)
Cleavage	14 (50)	4 (31)	9 (27)	10 (48)
No cleavage	14 (50	9 (69)	26 (73)	11 (52)

*a* Number in brackets denotes percentage (%)

### Relationship between 3xGly cleavage and virulence

**Figure 4:**
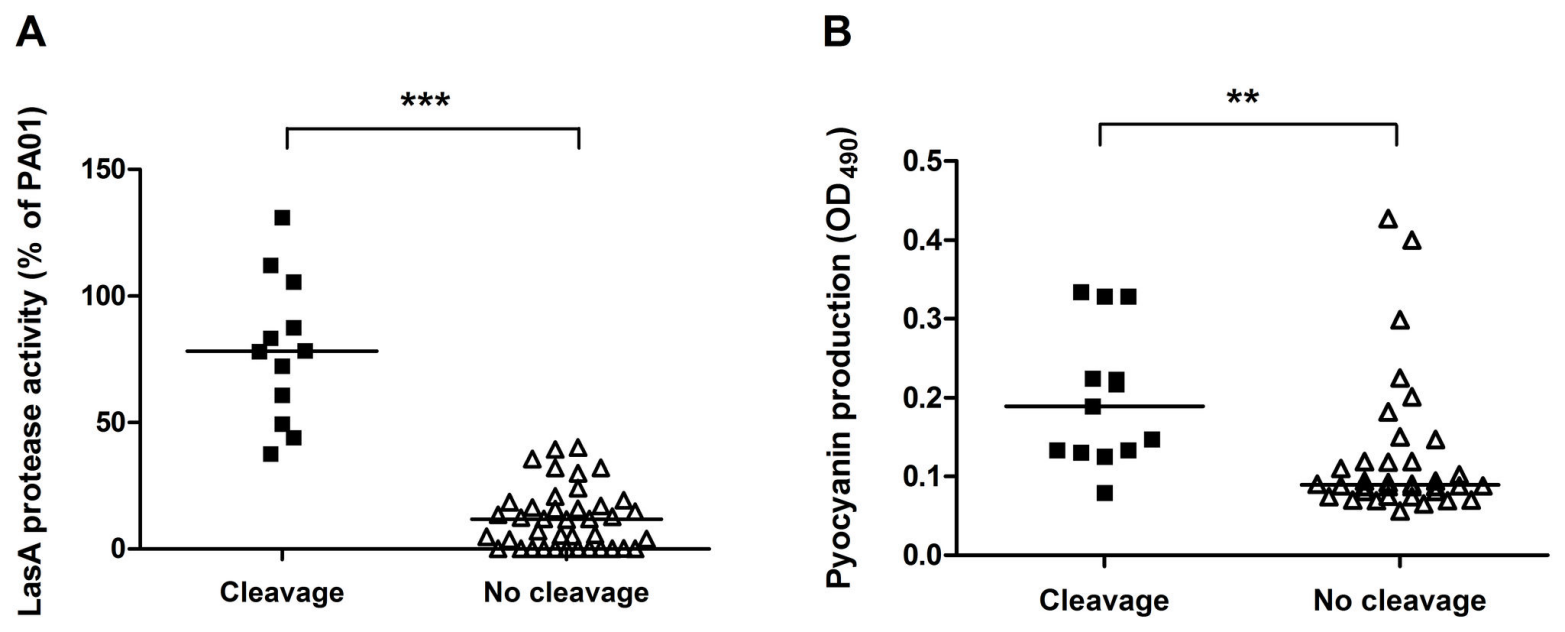
Link between 3xGly cleavage and secretion of QS-related proteins. Culture supernatants of 56 *P. aeruginosa* strains isolated from sputum were analyzed for LasA protease activity (A) and pyocyanin production (B). QS-related LasA cleavage activity in 3xGly cleaving *P*. *aeruginosa* strains (RF/min > 5) was compared to LasA cleavage in 3xGly non-cleaving strains (RF/min < 5) using the unpaired, two-tailed students *t*-test (** *P* < 0.01, *** *P* < 0.0001). A horizontal line indicates the median LasA activity /pyocyanin production.

LasA is a member of the beta-lytic endopeptidase family and pyocyanin is a secondary metabolite which has the ability to oxidize and reduce other molecules [21,22]. The expression of both of these virulence factors by *P. aeruginosa* has been shown to be related to its QS system [2]. Results from both LasA and pyocyanin production indicated that both were significantly higher in 3xGly cleaving *P. aeruginosa* isolates, though some variation in individual isolates was observed (Figure 4A-B). The association between LasA activity and 3xGly cleavage was very significant (*P* < 0.0001, *r* = 0.542). 

### Relationship between 3xGly cleavage and the quorum sensing system

**Figure 5:**
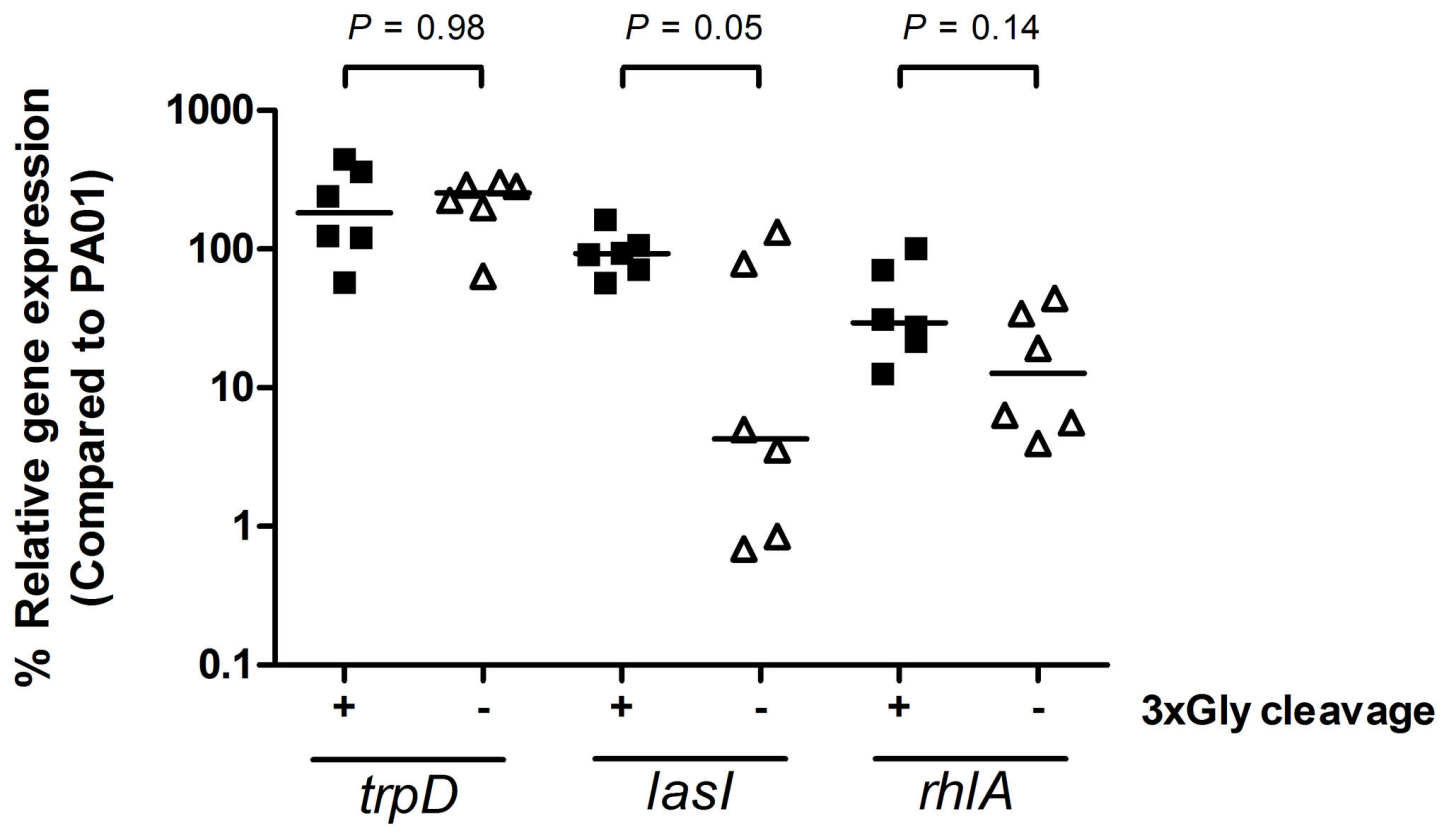
Link between 3xGly cleavage and expression of QS-genes. QS-gene expression was determined in a subset *P. aeruginosa* strains as described in material and methods. Normalized expression of the QS-circuit gene *lasI*, QS-target gene *rhlA* and the QS-independent gene *trpD* measured in mid-log phase grown bacteria is shown as relative values (%) compared to the P. *aeruginosa* PAO1 control strain. A horizontal line indicates the median expression levels. *P*-values were calculated using unpaired, two-tailed students *t*-tests.

The observation of a lack of 3xGly activity using a P. *aeruginosa lasI* knock-out mutant strain (Figure 1) indicated that an absence of 3xGly cleavage activity might be related to an impaired QS-system expression. In order to verify this hypothesis we investigated the expression of the QS-genes *lasI* and *rhlA* using a selection of 3xGly cleaving and non-cleaving *P. aeruginosa* isolates. The strains which lacked 3xGly proteolytic activity showed a reduced expression of *lasI* and *rhlA* when compared to *lasI* and *rhlA* expression in 3xGly cleaving isolates. A significant reduction in normalized *lasI*, but not *rhlA*, was observed (*P* = 0.05). There was no difference observed in the normalized expression of the *trpD* control, a gene unrelated to the QS-system (Figure 5). 

### Relationship between 3xGly cleavage and azithromycin susceptibility

**Figure 6:**
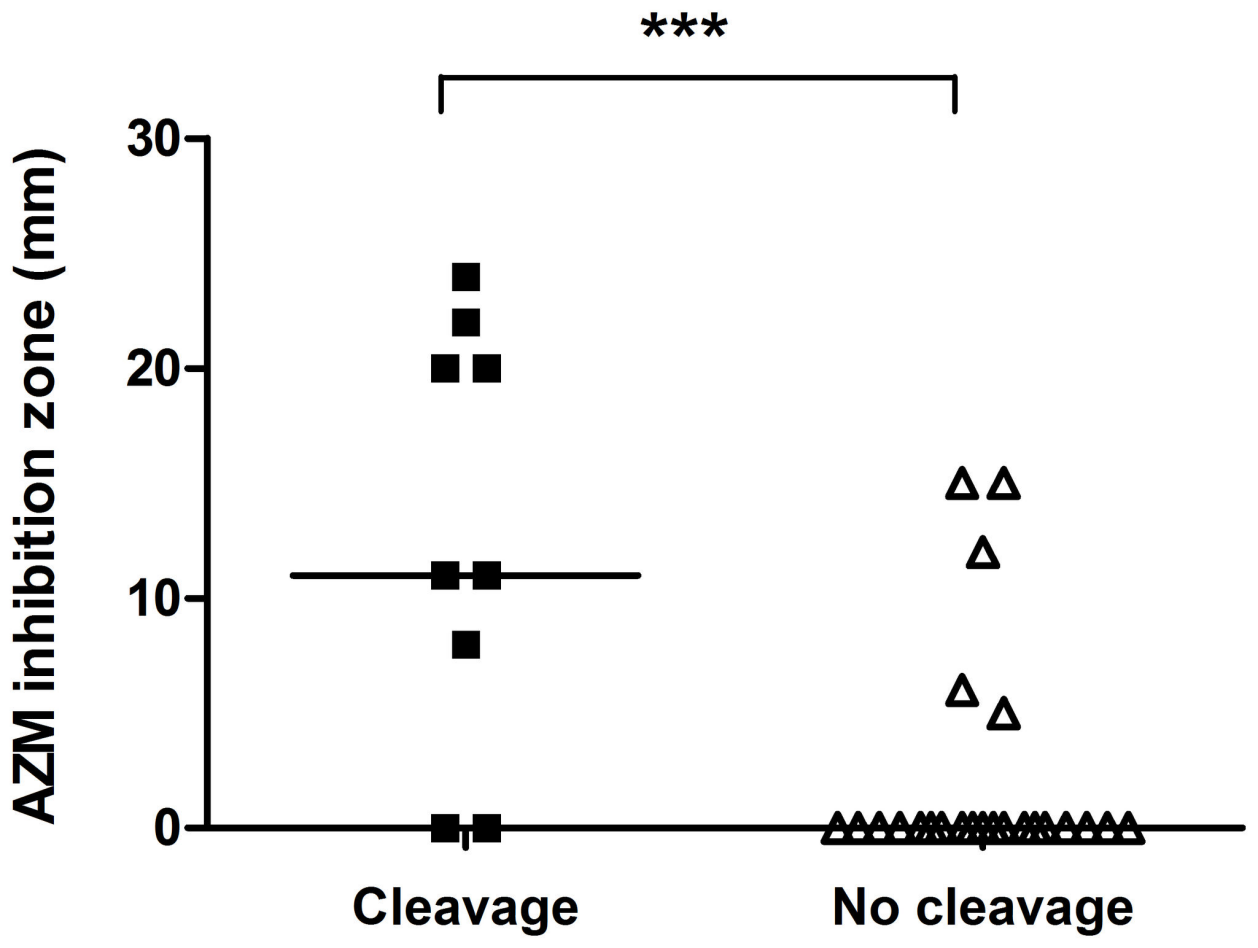
Link between 3xGly cleavage and azithromycin susceptibility. Azithromycin (AZM) susceptibility of *P. aeruginosa* isolates cultured from sputum was determined using the disc diffusion method. AZM inhibition zones for 3xGly cleaving (RF/min > 5) *P*. *aeruginosa* strains was compared to inhibition zones of 3xGly non-cleaving strains (RF/min < 5) using the unpaired, two-tailed students *t*-test (*** *P* < 0.0001). A horizontal line indicates the median AZM inhibition zone size.

 The apparent association between 3xGly cleavage and the QS-system led us to investigate a possible relationship between 3xGly cleavage and susceptibility to the QS-inhibiting antibiotic AZM. We examined the AZM susceptibility of *P. aeruginosa* clinical isolates using a disc diffusion assay, but found no relationship between 3xGly cleavage and AZM susceptibility for *P. aeruginosa* isolates cultured from blood and wounds, or from the sputum of non-CF patients. Interestingly however, a highly significant association was observed between AZM inhibition zone and 3xGly cleavage for *P. aeruginosa* isolates cultured from the sputa of CF patients (Figure 6). Finally, 3xGly cleavage in relation to a range of AZM unrelated antibiotic resistances was investigated, with no significant correlation being found between 3xGly cleavage and resistance to the other antibiotics tested. However, it was noted that among the group of *P. aeruginosa* isolates that lacked 3xGly cleavage more multi drug resistant (MDR) isolates were observed (Table S1).

## Discussion

We designed and evaluated a fluorogenic substrate as a potential marker of virulence in the bacterial pathogen *P. aeruginosa*. Preliminary experiments indicated that this 3xGly substrate was specific for cleavage by *P. aeruginosa*, and that the sensitivity of the substrate was 10^7^ CFU/ml within 1 h, though this limit of detection may vary among different *P. aeruginosa* strains (it was observed that PA14 cleaved the 3xGly substrate more efficient than PAO1). Only slight cross reactivity was observed with another bacterial protease, lysostaphin, which recognizes and degrades pentaglycin bonds [20]. Later experiments using a broader range of 97 clinical isolates showed that 3xGly cleavage activity differed between *P. aeruginosa* isolates, with a large percentage of the isolates being unable to cleave the 3xGly substrate. This is possibly related to difference in expression of the P. *aeruginosa* QS-system. We indeed observed a slight, but non-significant, decrease in expression of the QS-genes *lasI* and *rhlI* in *P. aeruginosa* strains which lacked 3xGly proteolytic activity. Because this experiment was performed with a subset of the P. *aeruginosa* strains future experiments are required in order to identify the exact genetic basis of the proteolysis / virulence correlation. 

One of the most important proteases secreted under the direction of the P. *aeruginosa* QS system is the LasA protease. The LasA protease (staphylolysin) of *P. aeruginosa* can recognize and degrade glycine bonds, degrade elastin and is an important contributor to the pathogenesis of this organism. LasA (20 kDa) is a member of the beta-lytic endopeptidase family of extracellular bacterial proteases, and possesses high-level staphylolytic activity [7]. Further, Elston et al. showed that the LasA protease is capable of the degradation of peptides in which three glycines were present [23]. Therefore, based on the already established glycine-cleaving proteolytic activity of the P. *aeruginosa* LasA protease, its staphylolytic activity, its control via the P. *aeruginosa* QS system, and the results observed for 3xGly cleavage, it appears that the LasA protease is most likely the protease virulence factor measured in *P. aeruginosa* culture supernatant using our 3xGly substrate, but this needs to be confirmed experimentally.

Other factors, dependent on the expression of the QS-system of *P. aeruginosa* are pyocyanin production and antibiotic resistance. Pyocyanin is an important virulence factor and functions as an electron transfer facilitator [2]. In addition, *P. aeruginosa* isolates which lack production of QS-dependent virulence factors, have previously been shown to possess a higher resistance rate to antimicrobials, among which ciprofloxacin and tobramycin [24]. Although, we indeed observed a significant correlation between pyocyanin production and 3xGly cleavage, no significant correlation with resistance to the antimicrobials examined was found (Table 1). This discrepancy might be due to difference in culture conditions, as the secretion of QS-metabolites, such as the LasA protease, depends on the availability of nutrients in the environment [25]. 

Perhaps one of the most interesting findings of the study was the fact that the highest percentage of 3xGly non-cleaving isolates was observed in group of CF sputum isolates. This is possibly due to the fact that CF patients are often colonized with *P. aeruginosa* [26]. Kohler et al showed that during *P. aeruginosa* colonization of intubated patients the number of QS- mutants increases. These mutants lack the production of QS-dependent proteins, such as elastase and rhamnolipids, and take advantage of the “public goods” produced by wild-type isolates that reside within the total population of *P. aeruginosa* isolates that colonize the patient (“cheater” strains). This phenomenon may result in overgrowth of the wild-type *P. aeruginosa* isolate by the mutant isolate due to its fitness benefit [27]. 

Currently, research is being performed to investigate the potential of QS-inhibiting compounds in the treatment of *P. aeruginosa* related infections [8–12]. The most thoroughly investigated QS-inhibiting antibiotic is AZM. AZM treatment is associated with improvement in disease outcome in *P. aeruginosa* infected CF-patients [28]. However, an anti-virulence agent such as AZM is only effective when an active, virulence factor secreting, QS-system is present. For this reason Kohler et al screened for rhamnolipid production to select patients for their clinical trial on AZM efficiency. Patients infected with *P. aeruginosa* strains which are unable to produce these QS-proteins were excluded from the protocol [12]. 

We observed a strong correlation between AZM susceptibility and 3xGly cleavage by *P. aeruginosa* isolates cultured from the sputum of CF patients, with growth inhibition zones in the 3xGly cleavage-positive isolates being significant larger compared to zones of 3xGly cleavage-negative isolates. Until recently it was stated that AZM is unable to eradicate *P. aeruginosa* by bacterial killing [29]. A recent publication by Buyck et al. however, revealed that growth inhibition of *P. aeruginosa* by AZM depends on the medium used [30]. *P. aeruginosa* strains grown in Mueller Hinton medium have a lower outer membrane permeability compared to strains cultured in for example RPMI. This results in an increase in susceptibility towards AZM and thus might explain the presence of AZM induced growth inhibition zones we observed on TSA agar plates.

In conclusion, we designed and evaluated a novel 3xGly FRET-peptide substrate for the assessment of virulence in *P. aeruginosa*. Cleavage of the 3xGly FRET-substrate was significantly associated with culture supernatant protease activity, staphylolytic activity, pyocyanin production and the expression of *lasI* in the P. *aeruginosa* QS system. This publication represents the first step in the development of a simple test to determine and monitor *P. aeruginosa* virulence in clinical samples, including the prediction of the effectiveness of QS-inhibiting antibiotic treatments. Preliminary evaluation experiments suggest that this methodology may achieve its greatest potential when monitoring CF patients colonized by, and undergoing treatment for, *P. aeruginosa* infections.

## Supporting Information

Table S1 **Antibiotic susceptibility of the 97 *P. aeruginosa* strains used in this study.**
(DOCX)
