# Supplementary material for: Evaluation of a FRET-Peptide Substrate to Predict Virulence in Pseudomonas aeruginosa
Source: PLoS One. 2013 Nov 26;8(11):e81428. doi: 10.1371/journal.pone.0081428 (PMC3841150; doi:10.1371/journal.pone.0081428)
Supplement: Table S1 — Antibiotic susceptibility of the 97 P. aeruginosa strains used in this study. (DOCX) [file pone.0081428.s001.docx]

| **Sample** | **3xGly cleavage** | **Specimen** | **MER** | **IMP** | **CAZ** | **TOB** | **AMI** | **CIP** | **NOR** | **PIP** | **TAZ** | **ATM** | **COT** | **COL** |
| --- | --- | --- | --- | --- | --- | --- | --- | --- | --- | --- | --- | --- | --- | --- |
|  |  |  |  |  |  |  |  |  |  |  |  |  |  |  |
| B1 | - | Blood | S | S | S | R | S | R | R | n/t | n/t | n/t | n/t | n/t |
| B2 | - | Blood | R | R | R | R | n/t | R | R | R | R | n/t | n/t | S |
| B3 | - | Blood | S | S | S | S | S | S | S | n/t | n/t | n/t | n/t | n/t |
| B4 | - | Blood | S | S | S | S | S | S | S | n/t | n/t | n/t | n/t | n/t |
| B5 | - | Blood | n/t | S | I | S | S | S | S | S | n/t | S | n/t | n/t |
| B6 | - | Blood | S | S | S | S | S | S | S | n/t | n/t | n/t | n/t | n/t |
| B7 | - | Blood | S | S | S | S | S | S | S | I | I | n/t | n/t | S |
| B8 | - | Blood | S | R | R | R | I | R | R | n/t | n/t | n/t | n/t | n/t |
| B9 | - | Blood | S | S | S | S | S | S | S | I | I | n/t | n/t | S |
|  |  |  |  |  |  |  |  |  |  |  |  |  |  |  |
| S1 | - | Sputum | R | R | I | R | n/t | R | R | n/t | R | n/t | n/t | R |
| S2 | - | Sputum | n/t | S | S | S | S | S | S | S | S | n/t | R | n/t |
| S3 | - | Sputum | I | R | R | R | n/t | R | R | n/t | R | n/t | n/t | S |
| S4 | - | Sputum | n/t | R | R | R | R | I | R | I | I | n/t | R | n/t |
| S5 | - | Sputum | n/t | S | R | R | I | R | n/t | R | I | R | R | R |
| S6 | - | Sputum | I | R | R | R | n/t | R | R | n/t | R | n/t | n/t | S |
| S7 | - | Sputum | n/t | R | S | S | S | S | S | S | S | n/t | R | n/t |
| S8 | - | Sputum | R | R | R | I | n/t | S | S | n/t | n/t | n/t | n/t | S |
| S9 | - | Sputum | n/t | S | S | S | S | S | S | S | S | n/t | R | n/t |
| S10 | - | Sputum | 4 | R | R | I | R | I | S | R | R | n/t | n/t | S |
| S11 | - | Sputum | n/t | S | S | S | S | S | S | S | S | n/t | R | n/t |
|  |  |  |  |  |  |  |  |  |  |  |  |  |  |  |
| S22 | - | Sputum (CF) | S | S | S | S | R | S | S | S | S | n/t | S | S |
| S23 | - | Sputum (CF) | S | S | S | S | R | S | S | S | S | S | R | S |
| S24 | - | Sputum (CF) | n/t | R | R | R | R | S | S | R | R | R | R | S |
| S25 | - | Sputum (CF) | n/t | S | S | S | S | R | R | S | S | S | R | S |
| S26 | - | Sputum (CF) | n/t | S | S | S | S | S | S | S | S | S | R | S |
| S27 | - | Sputum (CF) | n/t | S | S | R | R | R | R | S | S | S | R | S |
| S28 | - | Sputum (CF) | S | S | S | S | R | R | R | S | S | n/t | R | S |
| S29 | - | Sputum (CF) | n/t | S | S | S | S | S | S | S | S | S | R | S |
| S30 | - | Sputum (CF) | n/t | R | S | S | I | S | S | S | S | S | R | S |
| S31 | - | Sputum (CF) | R | S | R | R | R | S | I | S | S | R | R | R |
| S32 | - | Sputum (CF) | R | R | S | S | S | R | R | S | S | R | R | S |
| S33 | - | Sputum (CF) | n/t | S | R | R | R | R | R | R | R | R | R | S |
| S34 | - | Sputum (CF) | n/t | S | S | I | R | S | R | S | S | S | R | S |
| S35 | - | Sputum (CF) | S | S | S | S | S | S | S | S | S | S | R | S |
| S36 | - | Sputum (CF) | S | R | S | S | S | I | R | S | S | S | R | S |
| S37 | - | Sputum (CF) | n/t | S | S | S | S | S | S | S | S | S | R | S |
| S38 | - | Sputum (CF) | n/t | S | S | R | R | I | R | S | S | S | S | S |
| S39 | - | Sputum (CF) | S | S | S | S | S | S | S | S | S | S | R | S |
| S40 | - | Sputum (CF) | S | S | S | S | S | S | S | S | S | S | R | S |
| S41 | - | Sputum (CF) | S | R | S | S | S | I | R | S | S | S | R | S |
| S42 | - | Sputum (CF) | R | R | R | S | I | S | S | R | R | R | R | R |
| S43 | - | Sputum (CF) | S | S | S | S | S | S | S | S | S | S | R | S |
| S44 | - | Sputum (CF) | S | S | S | S | S | S | S | S | S | n/t | R | S |
| S45 | - | Sputum (CF) | R | R | I | S | n/t | S | S | n/t | I | n/t | R | S |
| S46 | - | Sputum (CF) | S | S | S | S | S | S | S | S | S | S | R | S |
| S47 | - | Sputum (CF) | n/t | R | R | R | R | R | R | R | R | R | R | S |
|  |  |  |  |  |  |  |  |  |  |  |  |  |  |  |
| W1 | - | Wound | S | S | S | S | n/t | S | S | n/t | S | n/t | R | S |
| W2 | - | Wound | S | S | S | S | S | S | S | n/t | n/t | n/t | n/t | n/t |
| W3 | - | Wound | R | R | 8 | R | n/t | R | R | n/t | 16 | n/t | n/t | S |
| W4 | - | Wound | S | S | S | S | n/t | S | S | n/t | S | n/t | R | S |
| W5 | - | Wound | R | R | R | R | n/t | R | R | n/t | n/t | n/t | n/t | S |
| W6 | - | Wound | S | S | S | S | n/t | S | S | n/t | S | n/t | R | S |
| W7 | - | Wound | I | R | R | R | S | I | S | S | R | n/t | n/t | n/t |
| W8 | - | Wound | R | R | R | R | n/t | R | R | n/t | R | n/t | n/t | S |
| W9 | - | Wound | S | S | S | S | n/t | S | S | n/t | S | n/t | R | S |
| W10 | - | Wound | I | I | R | R | S | I | S | I | R | n/t | n/t | S |
| W11 | - | Wound | R | R | R | R | n/t | R | R | n/t | R | n/t | n/t | S |
| W12 | - | Wound | S | S | I | I | n/t | I | I | n/t | R | n/t | n/t | S |
| W13 | - | Wound | S | S | S | S | n/t | S | S | n/t | S | n/t | R | S |
| W14 | - | Wound | S | S | R | R | n/t | R | R | n/t | n/t | n/t | n/t | S |
|  |  |  |  |  |  |  |  |  |  |  |  |  |  |  |

| **Sample** | **3xGly cleavage** | **Specimen** | **MER** | **IMP** | **CAZ** | **TOB** | **AMI** | **CIP** | **NOR** | **PIP** | **TAZ** | **ATM** | **COT** | **COL** |
| --- | --- | --- | --- | --- | --- | --- | --- | --- | --- | --- | --- | --- | --- | --- |
|  |  |  |  |  |  |  |  |  |  |  |  |  |  |  |
| B10 | + | Blood | R | R | S | R | R | R | R | n/t | n/t | n/t | n/t | n/t |
| B11 | + | Blood | S | S | S | S | S | R | R | I | I | n/t | n/t | S |
| B12 | + | Blood | R | R | R | R | R | R | R | n/t | n/t | n/t | n/t | n/t |
| B13 | + | Blood | R | R | R | R | I | R | R | R | R | R | n/t | S |
|  |  |  |  |  |  |  |  |  |  |  |  |  |  |  |
| S12 | + | Sputum | n/t | I | S | S | S | S | S | S | S | S | R | S |
| S13 | + | Sputum | S | S | S | S | S | S | S | S | S | n/t | R | n/t |
| S14 | + | Sputum | 1 | 2 | R | I | n/t | S | S | n/t | R | n/t | n/t | S |
| S15 | + | Sputum | R | R | S | S | n/t | S | S | I | I | n/t | R | S |
| S16 | + | Sputum | S | S | S | S | S | S | S | S | S | n/t | R | n/t |
| S17 | + | Sputum | 2 | R | R | R | R | R | R | R | R | R | n/t | S |
| S18 | + | Sputum | 2 | R | R | R | I | R | R | R | R | n/t | n/t | S |
| S19 | + | Sputum | n/t | S | S | S | S | S | S | S | S | n/t | R | n/t |
| S20 | + | Sputum | S | S | S | S | S | I | R | S | S | n/t | R | n/t |
| S21 | + | Sputum | S | S | S | S | S | S | S | S | S | n/t | R | n/t |
|  |  |  |  |  |  |  |  |  |  |  |  |  |  |  |
| S48 | + | Sputum (CF) | n/t | S | S | S | R | S | S | S | S | S | S | S |
| S49 | + | Sputum (CF) | n/t | S | S | S | R | S | S | S | S | S | R | S |
| S50 | + | Sputum (CF) | S | S | S | S | R | S | S | S | S | n/t | R | S |
| S51 | + | Sputum (CF) | n/t | S | S | S | S | S | S | S | S | S | R | S |
| S52 | + | Sputum (CF) | n/t | S | S | S | S | S | S | S | S | S | R | S |
| S53 | + | Sputum (CF) | n/t | S | S | S | S | S | S | S | S | S | R | S |
| S54 | + | Sputum (CF) | n/t | S | S | S | R | S | S | S | S | S | R | S |
| S55 | + | Sputum (CF) | S | S | S | S | S | S | S | S | S | S | R | S |
| S56 | + | Sputum (CF) | S | S | S | S | S | S | S | S | S | S | R | S |
|  |  |  |  |  |  |  |  |  |  |  |  |  |  |  |
| W15 | + | Wound | R | R | R | R | n/t | R | R | n/t | R | n/t | R | S |
| W16 | + | Wound | S | S | S | S | n/t | S | S | n/t | S | n/t | R | S |
| W17 | + | Wound | S | S | S | S | n/t | S | S | n/t | S | n/t | R | S |
| W18 | + | Wound | R | R | R | R | n/t | R | R | n/t | R | n/t | n/t | I |
| W19 | + | Wound | S | S | S | S | n/t | S | S | n/t | S | n/t | R | S |
| W20 | + | Wound | R | R | R | S | n/t | S | S | n/t | R | n/t | R | S |
| W21 | + | Wound | R | R | R | R | I | R | R | R | R | n/t | n/t | 2 |
| W22 | + | Wound | I | R | R | R | n/t | R | R | n/t | n/t | n/t | n/t | 2 |
| W23 | + | Wound | S | S | S | S | n/t | S | S | n/t | S | n/t | R | S |
| W24 | + | Wound | S | S | S | S | n/t | S | S | n/t | S | n/t | R | S |
| W25 | + | Wound | R | R | S | S | S | R | R | n/t | n/t | n/t | n/t | n/t |
| W26 | + | Wound | S | S | S | S | n/t | S | S | n/t | S | n/t | R | S |
| W27 | + | Wound | I | R | R | R | R | R | R | n/t | R | n/t | n/t | S |
| W28 | + | Wound | S | S | R | S | n/t | S | S | n/t | R | n/t | R | S |
|  |  |  |  |  |  |  |  |  |  |  |  |  |  |  |

*^a^* MER: meropenem, IMP: imipenem, CAZ: ceftazidime, AMI: amikacin, CIP: ciprofloxacin, NOR: norfloxacin, PIP: piperacillin, TAZ: piptazobactam, ATM: aztreonam, COT: cotrimoxazol, COL: colistine

**Table S1.** Antibiotic susceptibility of the 97 *P. aeruginosa* strains used in this study *^a^*
